# Comparing Free and Pedicled Flaps for Leg, Ankle and Heel Reconstruction: An Analysis of Outcomes, Complications and Flap Selection Considerations

**DOI:** 10.3390/medsci14020305

**Published:** 2026-06-11

**Authors:** Claudiu Ioan Filip, Radu Alexandru Ilieș, David Andraș, Alexandra Caziuc, George Călin Dindelegan

**Affiliations:** 1 Department of Plastic and Reconstructive Surgery, “Iuliu Hațieganu” University of Medicine and Pharmacy, 400012 Cluj-Napoca, Romania; filip.claudiu.ioan@elearn.umfcluj.ro; 2 First Surgical Unit, Emergency County Hospital Cluj, 400006 Cluj-Napoca, Romania; caziuc.alexandra@umfcluj.ro (A.C.); george.dindelegan@umfcluj.ro (G.C.D.); 3 Faculty of Medicine, “Iuliu Hațieganu” University of Medicine and Pharmacy, 400012 Cluj-Napoca, Romania; 4 Department of General Surgery I, “Iuliu Hațieganu” University of Medicine and Pharmacy, 400012 Cluj-Napoca, Romania

**Keywords:** heel, ankle, leg, defects, free flap, pedicled flap, limb salvage, microsurgery, lower extremity

## Abstract

**Background/Objectives**: Reconstruction of defects in the ankle, foot and heel is complex because of the limited availability of local tissue and multiple comorbidities like diabetes mellitus and peripheral vascular disease. Even though free and pedicled flaps are widely used, their comparative effectiveness remains incompletely defined. **Methods**: This study presents a narrative analysis of 21 studies. From each study, we extracted data related to flap type, characteristics of the patient, indications, and outcomes: flap survival, limb salvage, functional recovery and complications. **Results**: Free flaps were mainly used for the management of large, complex, infected, or weight-bearing plantar defects and generally showed high rates of survival (~95–97%) with good functional outcomes and limb salvage rates. On the other hand, pedicled flaps and perforator-based flaps were principally used for small-to-medium defects and showed comparable survival rates in selected cohorts (up to ~98–100%), although direct comparison is limited by differences in defect complexity and patient selection. Overall, the functional outcomes appeared comparable across techniques in appropriately selected patients. However, long-term complications, such as ulceration in weight-bearing heel regions, remained frequent (reported rates were up to 39–41% in some free flap series). Sensory recovery and vascular status were key elements of long-term success, often exceeding flap type in predicting outcomes. **Conclusions**: Both free and pedicled flaps are effective options for reconstructing lower limb defects when appropriately indicated. While pedicled flaps remain preferred for smaller defects and high-risk patients, free flaps are generally better suited for extensive and more complex defects. The outcomes are influenced by several factors: individualized reconstruction strategy, characteristics of the defects, vascular status and patient comorbidities.

## 1. Introduction

Soft tissue defects of the distal leg, ankle, and heel represent some of the most complex reconstructive challenges in contemporary plastic and reconstructive surgery. The anatomical characteristics of these regions impose several limitations on the reconstructive strategy, owing to limited local tissue availability, thin soft tissue coverage, poor elasticity, the exposure of tendons and bone, and the biomechanical requirements for weight-bearing durability [1,2,3,4,5]. Additionally, heel reconstruction implies the restoration of protective sensation and resistance to repetitive shear forces in order to decrease the risk of long-term ulceration and optimize ambulation [1,3,4].

A free flap consists of a tissue transfer that is fully detached from its original vascular supply and subsequently revascularized at the recipient site via microvascular anastomosis. A pedicled flap, on the other hand, maintains its original vascular pedicle and is transferred to the site of the defect without the need to perform a microvascular anastomosis [2,3,4,5,6].

Historically, several heel and ankle defects led to amputation because reconstructive techniques were limited. The evolution of microsurgery and free tissue transfer expanded the reconstructive possibilities and allowed for limb salvage in many cases: extensive trauma, oncologic defects, diabetic ulceration, osteomyelitis and chronic infection [2,4,6,7]. Free flaps like the anterolateral thigh (ALT), thoracodorsal artery perforator (TDAP), latissimus dorsi, scapular and lateral arm flaps offer large tissue volumes, flexibility in flap design, and the possibility to reconstruct complex 3D defects [2,6,7,8,9]. Modern microsurgical techniques have shifted reconstructive paradigms from amputation toward functional preservation of limbs.

However, free flaps have the following disadvantages: they require advanced microsurgical expertise, extensive operative times, postoperative monitoring of the flap and intensive perioperative care, which are factors that consequently increase healthcare costs [4,6,10]. Furthermore, patients who undergo lower extremity reconstruction usually present with diabetes mellitus, peripheral vascular disease, a history of smoking, chronic kidney disease, and malnutrition (or obesity), all of which increase the risk of developing perioperative complications and flap loss [4,10,11,12]. Consequently, other flaps such as local, pedicled and perforator-based flaps continue to play an essential role in lower extremity reconstruction.

Pedicled flaps like reverse sural artery flaps, anterior tibial artery perforator flaps, peroneal artery perforator flaps, medial plantar artery flaps and random-pattern local flaps offer several advantages [1,5,11,12,13,14,15,16]. None of these techniques requires microsurgical anastomosis, which reduces operative complexity and potentially lowers hospitalization and resource utilization. Excellent coverage for small and medium-sized defects can be provided. Pedicled options are good options for patients with significant comorbidities, elderly patients and in healthcare systems with limited microsurgical infrastructure [1,5,13,14,15,16].

**Figure 1:**
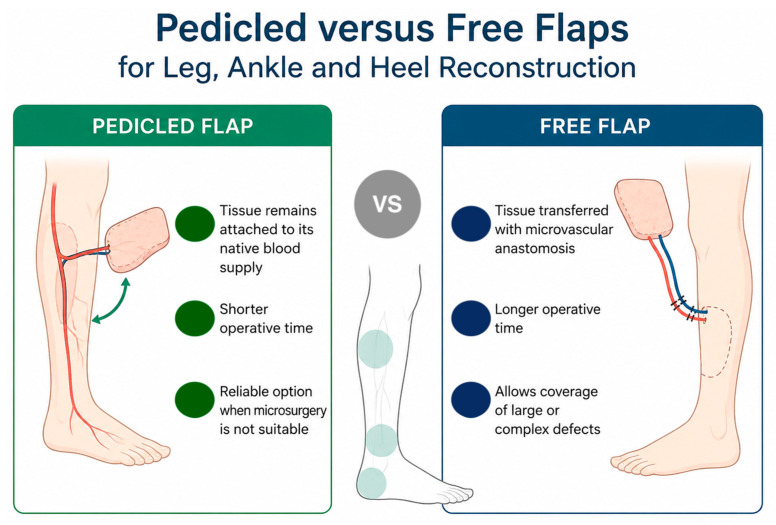
Schematic comparison between pedicled and free flaps used in lower limb reconstruction.

However, controversy regarding the optimal reconstructive strategy for distal lower extremity defects persists. Several studies advocate for the use of free flaps for the most complex heel defects due to their superior versatility [2,4,6,7], while others highlight the reliability and lower morbidity of pedicled flaps for appropriate indications [1,5,11]. The literature also reports challenges concerning ulcer recurrence, gait abnormalities, impaired sensation, donor-site morbidity, and even delayed amputation following reconstruction [1,4,10,12]. Reconstructive decisions depend on the surgical team’s experience, institutional expertise, the characteristics of the defect and patient-related factors (Figure 1).

The aim of this review is to analyze the existing evidence regarding free versus pedicled flaps for the reconstruction of leg, ankle and heel defects. Several factors represent our study’s focus, such as indications for each technique, flap survival, complication profiles, ulceration rate, sensory recovery, ambulation outcomes and patient-reported outcomes. Additionally, this review has the objective to synthesize current reconstructive algorithms based on the consulted literature.

## 2. Materials and Methods

The current study represents a structured narrative review intended to provide a clinically oriented synthesis of the current literature rather than a formal systematic review or meta-analysis. It compares the use of free and pedicled flaps for the reconstruction of distal lower extremity defects involving the ankle, heel, and foot.

We performed a structured search in the PubMed database using the following search strategy: “lower limb reconstruction” AND (“pedicled” OR “free”) AND “flap”. Additionally, the reference lists of the included articles were manually screened to find further studies. The search strategy was designed to identify clinically relevant studies evaluating reconstructive flap options for distal lower extremity defects. Because this review was narrative in nature, additional databases and formal systematic review methodology were not applied. The literature search was performed in May 2026.

Studies evaluating free or pedicled flap reconstruction for distal lower extremity defects involving the heel, ankle, or foot were considered suitable for our narrative review. Case reports, case series, retrospective and prospective studies were eligible for inclusion. Articles not written in English, studies without clinical outcomes, and studies unrelated to reconstructive flap surgery were excluded. Following the selection process, a total of 21 articles (out of 143 results) were extracted and included in the review. From each study, we extracted the following parameters, when available:

1. defect characteristics
2. flap type and reconstructive strategy
3. flap survival and complication rates
4. limb salvage outcomes
5. functional outcomes and ambulation
6. sensory recovery
7. ulceration and long-term outcomes
8. donor-site morbidity

## 3. Results

### 3.1. Analysis of the Included Studies

The 21 extracted studies consisted of a heterogeneous body of evidence, predominantly composed of retrospective studies (including cohorts, retrospective case series, and retrospective algorithmic analyses). Furthermore, the review included comparative studies, database studies, prospective cohorts, case reports, and case series, as well as one meta-analysis combined with a case series (Table 1).

Across the studies, patient populations were mainly composed of adults with significant comorbidities. Reported age ranges typically included middle-aged to elderly patients, with several studies focusing specifically on diabetic or vascular-compromised cohorts. The most frequently reported comorbidities included diabetes mellitus, peripheral vascular disease, a smoking history, renal dysfunction, and hypoalbuminemia, all of which were consistently associated with worse reconstructive outcomes and higher complication rates, as presented in Table 1 and further discussed in the subsequent sections.

Krishna et al. (2021) [1] performed a retrospective algorithm-based study, which included 40 patients with heel defects, with traumatic, neuropathic and chronic ulcer origins. Based on the defect characteristics, the reconstruction was realized using local/pedicled and free flaps. Flap survival rate was high, with only two reported cases of marginal necrosis. Delayed ulceration was present in six cases, predominantly in patients with neuropathic heels. Neuropathic patients had significantly worse sensory recovery, with lower functional scores in comparison to non-neuropathic cases [1].

Zhu et al. (2013) [2] provided a large retrospective series consisting of 226 foot and ankle reconstructions, including 164 free and 62 pedicled flaps. Overall, the survival of flaps was high, with eight reported complete free flap losses, without compromising limb preservation. Protective sensation was recovered in most of the patients (within 3–12 months). Plantar weight-bearing reconstructions showed delayed ulceration and infection in a subset of cases, highlighting long-term biomechanical vulnerability [2].

Eun and Woo (2022) [3] carried out a retrospective study, assessing various free flaps for reconstructing heel soft tissue in 13 patients: eight anterolateral thigh flaps, two lateral arm flaps, two superficial circumflex iliac artery perforator flaps and one temporalis muscle flap. High flap survival rates, together with good functional outcomes, were achieved across the different types of flaps. Complications such as partial necrosis, wound dehiscence, and bulky flap occurred in three cases, requiring minor revision surgery. Overall, patient satisfaction was high [3].

Sayyed et al. (2022) [4] conducted a retrospective study with 44 patients who underwent reconstruction with a free flap for plantar weight-bearing heel defects. The rate of microsurgical success was 95.6%, with a limb salvage rate equal to 73.3% at medium-term follow-up. More than 90% of patients remained ambulatory at the most recent follow-up. Factors like the presence of peripheral vascular disease, hypoalbuminemia, and postoperative infection represented significant predictors for the failure of limb preservation strategies [4].

Berger et al. (2025) [5] performed a retrospective cohort study of 206 patients who underwent local flap reconstruction for chronic ankle and foot wounds. Flap success rate was equal to 98.1%, with low rates of reintervention. Even if the initial coverage was good, 21.8% of the operated patients later required ipsilateral amputation. Approximately 73% of them remained ambulatory at the follow-up. Conditions like diabetes and renal disease, vascular comorbidities, and risk factors such as smoking were linked to worse outcomes.

Grauberger et al. (2020) [6] carried out a retrospective cohort study, which was based on 43 patients who were treated with free flap reconstruction for weight-bearing heel and Achilles tendon defects. Flap survival was achieved in 95.3% of cases, and limb salvage in 93% of cases. Functional outcomes were favorable, with most patients regaining independent ambulation (no or little impairment in 76.2% of cases, ability to walk half a mile or more in 85.7% of cases) and returning to work (86.6%). Ulceration occurred significantly more in weight-bearing heel reconstructions in comparison with Achilles region defects (41.7% vs. 6.5%) [6].

Gu et al. (2017) [7] described a clinical series with 11 patients undergoing reconstruction for heel defects with the use of a medial plantar artery island flap. The cause of the defect was trauma (two cases), calcaneal osteomyelitis with soft tissue infection (two cases) and stage IA melanoma (seven cases). All of the flaps survived successfully. Patients achieved good sensory recovery, with a mean two-point discrimination 34.4 mm at the heel and 17.2 mm at the distal sole, respectively. The esthetic satisfaction rate was high, with a mean score of 9. The flap provided durable, glabrous skin with good biomechanical compatibility for weight-bearing. The authors acknowledged limitations like restricted flap size and donor-site constraints [7].

Kang et al. (2013) [8] conducted a retrospective study on 49 patients with lower extremity reconstruction, with the use of free flaps for complex defects, focusing on functional recovery and wound coverage. The most commonly used flaps included anterolateral thigh and latissimus dorsi flaps. Free tissue transfer proved effective for covering exposed bone, tendon, and infected wounds; the global survival rate of the flaps was equal to 96.2%. Limb salvage outcomes were favorable in complex cases which required extensive reconstruction. However, complications occurred in 19 cases and were partial graft loss in eight cases, partial flap necrosis in six cases and infection in five cases [8].

Liaghat and Shabbooie (2025) [9] published a case series of four patients, in which they described sensate medial plantar free flap reconstruction with nerve coaptation in heel defects. In all cases, patients achieved good sensory recovery and stable ambulation. No ulceration or major complications were reported by the authors. The reconstructed tissue closely matched native heel characteristics, not only in terms of appearance, but also function [9].

Kim et al. (2020) [10] performed a retrospective study of 37 patients with free flap reconstruction for diabetic heel defects. Overall, the flap survival rate was 73%, significantly lower than in non-diabetic populations. Vascular insufficiency, particularly impairment of both arterial branches in the heel, was strongly linked to flap failure. Other risk factors included higher ASA class and infection. A high-level amputation was realized in a significant subset of patients following flap loss [10].

Lee et al. (2024) [11] carried out a comparative study of instep compared to non-instep flaps for weight-bearing forefoot and heel reconstruction in 39 cases. No significant differences were observed regarding the functional scores, pain, ulcer recurrence, or the complication rates. The authors concluded that flap selection ought to rely on defect characteristics and vascular status rather than the expected superiority of instep tissue [11].

Sakarya et al. (2022) [12] retrospectively analyzed 74 free flap reconstructions for weight-bearing heel defects in 70 patients. Flap survival was high (97%), with no statistically significant differences between the types of flap (muscle vs. fasciocutaneous flaps), nor the timing of the reconstruction. Ulceration occurred in 39% of cases, but ulcers frequently developed several years following reconstruction. Secondary contour revisions were commonly required to improve footwear compatibility and gait function [12].

Ahn et al. (2015) [13] performed a retrospective assessment of peroneal artery perforator-based pedicled flaps for reconstructing ankle and heel defects, based on 12 cases. All flaps survived, with minimal complications and low donor-site morbidity (in 2 cases, wound dehiscence occurred). The technique allowed for coverage of complex defects, while preserving the major vessels of the leg. Functional outcomes were favorable, as none of the patients had functional deficits [13].

Lin et al. (2020) [14] published a study analyzing a clinical series of 12 patients, in which they evaluated the use of anterior tibial artery perforator-based propeller flaps for the reconstruction of ankle and heel defects. Flap survival was complete in almost all of the cases (with only minor wound complications). Patients were capable of full ambulation with standard footwear and minimal functional impairment. The technique provided thin, pliable coverage, with decreased donor-site morbidity and preserved major vascular axes [14].

Li et al. (2024) [15], in their retrospective cohort study based on 204 patients, compared pedicled (in 118 cases) and random-pattern local flaps (in 86 cases) for reconstructing chronic foot and ankle defects. Random flaps were preferred mostly for superficial defects, whereas pedicled flaps were used for deeper wounds that had exposed structures. Both approaches achieved comparable long-term limb salvage outcomes. Pedicled flaps had higher rates of ischemic complications but remained effective in appropriately selected cases.

Sarker et al. (2024) [16] analyzed a clinical series of 31 patients who underwent reconstruction using reverse sural artery flaps with technical modifications. Most of the flaps survived completely (with 3 exceptions). Partial necrosis, marginal necrosis and epidermolysis occurred in a minority of cases, with each complication observed in a single patient. Overall, the method provided reliable coverage for posterior heel and ankle defects, even if venous congestion remained the main complication, particularly in high-risk patients [16].

Laitonjam et al. (2023) [17] conducted a prospective study of 50 patients who underwent local, pedicled, and free flap reconstruction for foot and ankle defects. Global flap survival was high, as only one free flap loss was reported. Functional and esthetic outcomes were equivalent between the techniques. Bulky flaps were less suitable for dorsal regions, while thicker flaps did better in weight-bearing areas. No significant differences regarding overall functional scores were observed between the groups [17].

Chellamuthu et al. (2023) [18], in their study (meta-analysis and case series) including 255 patients with 263 flaps, compared muscle and fasciocutaneous free flaps for heel reconstruction. No significant differences were found in terms of flap survival, ulceration rates, gait abnormalities, or reinterventions. Fasciocutaneous flaps showed increased sensory recovery in comparison with muscle flaps. Still, evidence quality was limited with statistical uncertainty regarding several outcomes [18].

Nageeb et al. (2025) [19], in their case report, described the use of combined medial plantar artery and chimeric thoracodorsal artery perforator flaps for reconstructing extensive diabetic heel defect. The patient achieved complete wound healing and regained ambulation. No flap loss or major complications occurred. The hybrid approach allowed for the achievement of both durable plantar coverage and large soft tissue volume [19].

Bernuth et al. (2024) [20] conducted a large database study on 6475 patients, analyzing the predictors of complications after performing pedicled lower extremity flap reconstruction. Major complication risk was linked to a higher ASA class, hypertension, extended operative time, and an inpatient setting. Bleeding and transfusion were the most common postoperative complications (occurring in 19.9% of cases). The study proposed a nomogram for preoperative risk stratification and patient counseling [20].

Ciofu et al. (2017) [21] showed in their clinical series study that the reverse sural flap is an effective option for ankle and heel soft tissue reconstruction, achieving successful defect coverage in all 10 patients included in their study. Although minor complications occurred in approximately 30% of cases, complete defect coverage was achieved without major flap loss. Functional outcomes were reported as very good, with acceptable esthetic results. The authors found the reverse sural flap to represent a reliable alternative to microsurgical free flap reconstruction in selected patients [21].

### 3.2. Flap Survival

Flap survival rates were consistently high across both free and pedicled flaps studies (Table 2).

Several studies reported flap survival exceeding 90%, mostly in non-diabetic populations. Pedicled flaps frequently demonstrated similarly high reported survival rates in selected cohorts compared to free flaps, despite the substantially lower operative complexity [5,13,16]. However, direct comparison is limited by differences in defect complexity and patient selection. Flap survival was reported to be lower in diabetic patients with severe vascular disease [10]. Several studies emphasized that flap survival alone does not necessarily predict long-term functional success or limb preservation [4,5,10].

### 3.3. Limb Salvage

Free flap reconstruction achieved excellent limb salvage outcomes in complex heel defects. Patients undergoing free tissue transfer frequently presented with complex defect etiologies, including trauma, chronic infection, diabetic ulceration, and a previously failed reconstruction. These cases were commonly associated with systemic risk factors such as diabetes mellitus and peripheral vascular disease, which influenced both limb salvage rates and postoperative complication profiles.

Limb salvage outcomes were generally favorable across the included studies. Reported limb salvage rates ranged from 73.3% in the study by Sayyed et al. [4] to 93% in the cohort analyzed by Grauberger et al. [6]. Zhu et al. [2] reported very high limb preservation rates despite a small number of flap losses, while Sakarya et al. [12] and Kang et al. [8] also described high and favorable limb salvage outcomes following reconstruction. In the studies, free tissue transfer proved to be particularly effective for:

- large plantar defects
- an exposed calcaneus
- chronic osteomyelitis
- traumatic tissue loss
- a failed previous reconstruction

Microsurgical reconstruction has considerably expanded limb preservation possibilities in patients who historically might have required amputation [2,4,6]. 

Pedicled flaps also showed strong limb preservation outcomes [5,13,16]. Berger et al. reported that despite successful local flap reconstruction, 21.8% of patients eventually progressed to an ipsilateral amputation [5]. However, this cohort included medically complex patients with chronic wounds, diabetes and severe vascular disease.

### 3.4. Functional Outcomes and Ambulation

Functional outcomes were also influenced by patient-related factors. Globally, younger patients and those without significant vascular disease achieved superior ambulation and functional recovery, while diabetic and neuropathic patients demonstrated reduced sensory recovery and a higher risk of long-term gait impairment.

Grauberger et al. reported particularly strong functional outcomes after free flap reconstruction [6]: 85.7% of patients walked at least half a mile, 86.6% returned to work, and 76.2% experienced little or no daily functional impairment. Similarly, Sayyed et al. reported that more than 90% of patients were ambulatory after plantar heel free flap reconstruction [4].

Lin et al., in their study, stated that patients reconstructed with anterior tibial artery perforator flaps regained ambulation with standard footwear and a minimal functional deficit [14]. Berger et al. reported ambulation in approximately 73% of patients after local flap reconstruction [5]. The prospective study performed by Laitonjam et al. found no major differences in terms of functional scores between the local and free flap groups [17].

### 3.5. Sensory Recovery

Several free flap studies demonstrated favorable sensory recovery. Zhu et al. reported recovery of protective sensation in all but one case within 3–12 months postoperatively [2]. Liaghat and Shabbooie reported excellent sensory recovery after sensate medial plantar free flap reconstruction with nerve coaptation [9]. Chellamuthu et al. demonstrated that fasciocutaneous free flaps provided significantly improved sensory recovery (in terms of light touch sensation, pain perception and deep pressure sensation) compared with muscle flaps [18].

Pedicled sensate flaps such as the medial plantar artery flap also showed encouraging sensory outcomes [7]. Gu et al. reported restoration of protective sensation and good esthetic outcomes after a medial plantar artery flap reconstruction [7]. Krishna et al. stated that neuropathic patients experienced significantly poorer sensory recovery than non-neuropathic patients regardless of flap type [1].

### 3.6. Ulceration and Long-Term Durability

The development of late ulceration was more frequently noticed in patients with diabetes mellitus, peripheral neuropathy, and peripheral vascular disease, suggesting that systemic comorbidities play a critical role in long-term flap durability beyond the choice of reconstructive technique.

Several studies reported high ulcer recurrence rates after free flap reconstruction. Postoperative ulceration remained a relevant long-term complication across several studies. Krishna et al. [1] reported ulceration in six patients out of 40, while Zhu et al. [2] identified six cases of plantar flap ulceration out of 32 free flaps for plantar foot coverage. High ulceration rates were particularly observed in weight-bearing heel reconstructions, with Grauberger et al. [6] reporting ulceration in 41.7% of cases and Sakarya et al. [12] describing an overall ulceration rate of 39%. Moreover, Sakarya et al. stated that ulceration might occur several years after reconstruction, emphasizing the need for a prolonged follow-up [12].

Pedicled flaps also demonstrated ulcer recurrence, even if rates were less consistently reported, as Krishna et al. [1] mentioned that delayed ulceration occurred in both locoregional and free flap groups. Zhu et al. [2] identified five cases of plantar flap ulceration from the total of 25 pedicled flaps for plantar foot coverage.

### 3.7. Donor-Site Morbidity

Free flap donor-site morbidity varied according to flap type. ALT flaps generally provided favorable donor-site outcomes because primary closure is frequently possible [2,4,6]. Latissimus dorsi flaps may cause shoulder weakness, seroma formation and larger scars, while scapular and TDAP flaps generally demonstrated acceptable donor-site morbidity but require repositioning during surgery.

Pedicled flaps often involved lower donor-site morbidity. Perforator flaps preserved major arteries and muscle function [13,14]. Despite these, reverse sural flaps might cause sensory deficits, venous congestion, distal necrosis and a bulky contour deformity. Medial plantar flaps might create plantar donor-site discomfort or delayed healing [7].

## 4. Discussion

### 4.1. Overview of the Main Findings in the Analyzed Studies

Distal lower extremity reconstruction is still one of the most technically demanding and biologically complex fields in plastic and reconstructive surgery. The current review shows that both free and pedicled flaps have demonstrated high reported flap survival rates when appropriately selected (depending on defect characteristics and patient-related factors), with satisfactory limb salvage and functional recovery [1,2,3,4,5,6,7,8,9,10,11,12,13,14,15,16,17,18,19,20].

The reviewed literature does not clearly demonstrate the superiority of one reconstructive strategy over another; however, comparisons are limited by strong selection bias, as free flaps are preferentially used for larger and more complex defects. Instead, successful reconstruction relies on individualized decision-making that integrates anatomical, vascular, systemic and biomechanical factors.

One of the clearest findings across the reviewed studies was the dominant role of free flap reconstruction in extensive plantar and weight-bearing heel defects [2,4,6,8,10,12]. Large defects with an exposed calcaneus or Achilles tendon, and those with osteomyelitis frequently exceed the reconstructive capacity of local tissues and, thus, require microsurgical free tissue transfer. Free flaps provide vascularized tissue that can obliterate dead space, improving local perfusion and restoring soft tissue bulk in wounds which are severely compromised [2,6,8].

The anterolateral thigh flap emerged as one of the most versatile and frequently utilized options throughout the literature [2,4,6,8,12]. Its advantages include:

- a long vascular pedicle
- a large skin paddle
- a relatively low donor-site morbidity
- the ability to harvest sensate components
- the potential for thinning and contour modification

Likewise, latissimus dorsi musculocutaneous flaps were particularly valuable in infected wounds and extensive dead-space defects due to their large tissue volume and robust vascularity [2,8].

Patients who undergo lower extremity reconstruction commonly present with diabetes mellitus, peripheral vascular disease, a smoking history, renal insufficiency and malnutrition, all of which increase perioperative risk and threaten flap survival [4,5,10,20].

Kim et al. illustrated the devastating impact of vascular compromise in diabetic heel reconstruction, stating that impairment of both heel arterial branches increased the flap failure risk by approximately 80-fold [10]. These findings support the principle that successful reconstruction relies not only on flap selection but also on the optimization of systemic and vascular conditions prior to surgery.

At the same time, the reviewed studies clearly show that pedicled and perforator-based flaps remain extremely valuable reconstructive options [1,5,13,14,15,16]. Reverse sural artery flaps, peroneal artery perforator flaps, anterior tibial artery perforator flaps and medial plantar artery flaps obtained excellent outcomes with lower operative complexity and reduced resource utilization.

Perforator-based reconstruction is one of the most important modern advances in lower extremity reconstruction. Studies by Ahn et al. and Lin et al. showed that perforator flaps provide reliable vascularity, while preserving major vascular trunks and decreasing donor-site morbidity [13,14]. Moreover, these flaps provide thinner and more pliable tissue than many free flaps, improving contour and footwear compatibility around the ankle region.

The reverse sural artery flap is especially advantageous because of its technical simplicity and broad applicability [16]. Still, venous congestion, distal necrosis and sensory deficits remain important limitations. These complications become problematic mostly in smokers, diabetic patients and those with peripheral vascular disease.

One of the most important findings across several studies is that ulcer recurrence remains the principal long-term complication after heel reconstruction [1,2,4,6,12]. Even technically successful reconstruction can fail functionally due to the fact that the reconstructed tissue cannot fully replicate the biomechanical properties of native plantar heel tissue.

Sakarya et al. reported ulceration rates approaching 39% after free flap heel reconstruction [12], while Grauberger et al. demonstrated significantly higher ulcer recurrence in weight-bearing heel reconstructions compared with Achilles region defects [6]. Ulceration frequently developed several years following surgery, emphasizing the necessity for prolonged surveillance and long-term patient education.

The reviewed evidence suggests that sensory recovery plays a critical role in preventing ulceration and improving durability [1,7,9,18]. Fasciocutaneous free flaps appear to show improved sensory recovery in comparison with muscle flaps [18]. Likewise, medial plantar artery flaps and sensate free flaps with nerve coaptation obtained favorable long-term outcomes, as they restored protective sensation [7,9].

Furthermore, the superiority of sensate reconstruction remains incompletely established, as high-quality comparative studies are lacking; the meta-analysis and case series by Chellamuthu et al. indicated better sensory outcomes and an earlier weight-bearing after fasciocutaneous reconstruction, but found no significant differences regarding ulceration or overall flap survival between muscle and fasciocutaneous flaps [18].

An additional important issue involves the balance between durability and contour. Bulky flaps can provide superior resistance to pressure and shear forces, but at the same time they can impair footwear compatibility and gait [17]. Conversely, thin flaps improve contour, but might lack sufficient padding for plantar weight-bearing surfaces. Hence, flap thickness must be tailored according to the anatomical location and biomechanical demands.

### 4.2. Practical Algorithm for Flap Selection

Table 3 illustrates the preferred options for each type of defect, based on the conclusions of the analyzed studies.

#### 4.2.1. Small Superficial Defects

Small defects without exposed tendon or bone are often amenable to:

- local advancement flaps
- random-pattern local flaps
- medial plantar artery flaps
- small perforator flaps

These options minimize donor-site morbidity and operative burden while preserving satisfactory contour and ambulation [1,4,13,16].

#### 4.2.2. Medium-Sized Ankle and Achilles Defects

Medium defects involving the Achilles tendon region or ankle are commonly reconstructed using:

- reverse sural artery flaps
- anterior tibial artery perforator flaps
- peroneal artery perforator flaps
- propeller perforator flaps

These techniques provide reliable coverage with an acceptable contour, while lowering donor-site morbidity compared to free flaps [13,14,16].

Perforator-based pedicled flaps are advantageous because they preserve major vascular trunks while allowing the transfer of thin and pliable tissue into the defect [13,14].

#### 4.2.3. Large Plantar Weight-Bearing Defects

Large heel and plantar defects remain one of the main indications for microsurgical free flap reconstruction [2,4,6,12].

Preferred options include:

- an anterolateral thigh flap
- a latissimus dorsi musculocutaneous flap
- a thoracodorsal artery perforator flap
- a scapular flap
- sensate fasciocutaneous free flaps

Muscle flaps remain particularly valuable in infected wounds and extensive dead-space defects [2,8,18].

Sensate fasciocutaneous reconstruction ought to be considered whenever possible to improve protective sensation and reduce the ulceration risk [7,9].

#### 4.2.4. Diabetic and Neuropathic Heel Defects

Diabetic and neuropathic heel wounds represent some of the most challenging reconstructive scenarios because of peripheral arterial disease, neuropathy, impaired wound healing, chronic infection and increased ulcer recurrence risk.

The reviewed studies demonstrated that vascular optimization is essential before reconstruction [4,10,19]. Preoperative angiography, revascularization procedures and angiosome-guided debridement might significantly optimize outcomes in diabetic limb salvage.

Free flaps are usually necessary for extensive diabetic heel defects (because local tissue quality is often poor and defects are large) [4,10]. However, diabetic patients also show significantly higher complication and flap failure rates. Reconstructive planning in diabetic patients must include a careful vascular assessment.

Hybrid reconstructive approaches combining sensate plantar tissue with free tissue transfer may provide advantages in selected patients [19].

#### 4.2.5. Chronic Infection and Osteomyelitis

Chronic osteomyelitis and infected lower extremity wounds require an aggressive debridement and the obliteration of dead space.

Muscle free flaps remain highly valuable in these situations because they provide robust vascularity, dead-space obliteration, improved antibiotic delivery and acceptable infection control. Latissimus dorsi musculocutaneous flaps remain particularly useful for extensive infected defects [2,8]. Several authors stated that reconstructive success depends heavily on a complete debridement before flap coverage [2,8,10].

### 4.3. Key Claims and Level of Evidence

The evidence was qualitatively assessed according to the consistency of reported outcomes, study design, and reproducibility across the included studies (Table 4). The strength of evidence was then graded based on the predominance of cohorts, case series, and available comparative analyses, with a focus on consistency across studies rather than individual study size.

### 4.4. Limitations of the Current Study

The purpose of this review was to provide a clinically oriented synthesis of the current literature rather than a formal systematic review. Our review has several important limitations that should be acknowledged when interpreting the findings.

First, the included evidence is predominantly based on case series, cohort studies, and one meta-analysis with case series and a case report, with no randomized controlled trials directly comparing free versus pedicled flaps for ankle and heel reconstruction. As a result, the level of evidence is moderate, and strong causal inferences cannot be established.

Second, there is significant heterogeneity across the included studies with respect to patient populations, defect etiology and reconstructive indications. Included cases range from acute trauma to chronic diabetic ulcers, oncologic resections and infected wounds. This variability introduces a selection bias, because free flaps are often preferentially used for larger and more complex defects, while pedicled flaps are selected for smaller or less severe cases.

Third, outcome measures are inconsistent between the included studies. Flap survival and limb salvage rates, functional scores, patient-reported outcomes, ulceration rates, and follow-up durations are not uniformly reported. This limited our ability to perform meaningful pooled quantitative comparisons.

Fourth, the follow-up duration was highly variable and often insufficient to fully capture late complications. Some of the studies demonstrate that ulceration and functional deterioration might occur years after reconstruction, particularly in weight-bearing heel regions [6,12]. Short- and medium-term results can, though, overestimate long-term success.

Fifth, a publication bias is likely present, as studies reporting successful outcomes of both free and pedicled flaps are more likely to be published than negative or failed experiences. In addition, many studies originate from high-volume microsurgical centers, limiting the generalizability to low-resource settings.

Finally, important confounding factors like the severity of diabetes, peripheral vascular disease, nutritional status, a smoking and infection burden are not consistently controlled across these studies. All these parameters are known to strongly influence flap outcomes and might be more predictive of failure than the flap type itself.

## 5. Conclusions

Both free and pedicled flaps are reliable options for the reconstruction of ankle, heel, and foot defects when appropriately selected, relying on defect characteristics and patient comorbidities. The currently available literature does not clearly demonstrate the superiority of one technique over the other, although direct comparisons are limited by study heterogeneity and selection bias.

Free flaps are often preferred in large, complex, infected or weight-bearing defects, where they can provide superior tissue availability, reliable vascularity, and effective dead-space obliteration.

Pedicled and perforator-based flaps offer good outcomes in small to medium defects, with the advantages of lower morbidity and broader accessibility, making them valuable especially in elderly or medically fragile patients.

Across both reconstructive strategies, long-term complications (particularly ulceration in weight-bearing heel regions) remain the main limitation. These complications are strongly influenced by sensory recovery, neuropathy, vascular status, and biomechanical stress rather than the flap type itself.

Overall, the flap selection should be individualized within an algorithmic approach that integrates defect size, anatomical location, vascular condition, infection status and patient-specific risk factors. Future comparative studies are required to better define comparative effectiveness and long-term functional outcomes, together with the cost-effectiveness of reconstructive strategies.

## Figures and Tables

**Table 1:** Summary of included studies evaluating free vs. pedicled flaps in ankle, heel, and foot reconstruction.

Study	Design	Flap Types	Key Outcomes
Krishna et al. (2021) [1]	Retrospective algorithmic	Local + pedicled + free	High survival; 5% marginal necrosis; 15% delayed ulcerations; worse outcomes in neuropathic heels
Zhu et al. (2013) [2]	Retrospective series	164 free/62 pedicled	3.5% free flap losses; near-universal limb salvage; late plantar ulceration/infection
Eun and Woo (2022) [3]	Retrospective series	Various free flaps	High survival; good esthetic outcomes; minor revisions sometimes required
Sayyed et al. (2022) [4]	Retrospective cohort	Free flaps	95.6% success; 73.3% limb salvage; >90% ambulatory; vascular disease and hypoalbuminemia are predictive factors for the failure of limb salvage strategies
Berger et al. (2025) [5]	Large cohort	Local flaps	98.1% flap success; 21.8% amputation; 73% ambulatory; comorbidities strongly predictive
Grauberger et al. (2020) [6]	Retrospective cohort	Free flaps	95.3% survival; 93% limb salvage; high return-to-work; high ulceration in weight-bearing heel (41.7%)
Gu et al. (2017) [7]	Case series	Medial plantar flap	100% survival; excellent sensate reconstruction; high satisfaction
Kang et al. (2013) [8]	Retrospective series	Free flaps	Effective for complex trauma/infection; reliable limb salvage
Liaghat and Shabbooie (2025) [9]	Case series	Sensate medial plantar free flap	Excellent sensation; no ulceration; good functional recovery
Kim et al. (2020) [10]	Retrospective cohort	Free flaps (diabetic heel)	73% survival; high amputation risk; severe vascular disease key predictor
Lee et al. (2024) [11]	Comparative study	Instep vs. non-instep	No significant differences in function, pain, or ulceration
Sakarya et al. (2022) [12]	Retrospective series	Free flaps	High survival; 39% ulceration; late complications common
Ahn et al. (2015) [13]	Retrospective series	Peroneal perforator flaps	Complete survival; low morbidity; reliable ankle/heel coverage
Lin et al. (2020) [14]	Clinical series	Perforator pedicled	Near 100% survival; excellent ambulation; minimal complications; thin pliable coverage
Li et al. (2024) [15]	Retrospective cohort	Pedicled vs. random flaps	Similar long-term outcomes; pedicled higher ischemic risk
Sarker et al. (2024) [16]	Clinical series	Reverse sural flap	Majority survival; minor necrosis in few cases; venous congestion main complication
Laitonjam et al. (2023) [17]	Prospective cohort	Local + pedicled + free	Similar functional outcomes across groups; one free flap loss; no major differences in scores
Chellamuthu et al. (2023) [18]	Meta-analysis and Case series	Muscle vs. fasciocutaneous free flaps	Similar survival/ulceration; fasciocutaneous better sensation and earlier weight-bearing
Nageeb et al. (2025) [19]	Case report	Combined free flaps	Complete healing; good function; no major complications
Bernuth et al. (2024) [20]	Database study	Pedicled flaps	Complications linked to ASA, hypertension, time; bleeding most common; nomogram proposed
Ciofu et al. (2017) [21]	Prospective case series	Pedicled flaps	100% defect coverage; no major complications; ~30% minor complications (distal tip necrosis and transient venous congestion); good functional results; acceptable esthetic outcomes; reliable alternative to free flap reconstruction.

ASA = American Society of Anesthesiologists Physical Status Classification System [22].

**Table 2:** Summary of flap survival outcomes reported across the included studies.

Study	Flap Type	Flap Survival
Zhu et al. [2]	164 free + 62 pedicled flaps	High overall survival
Sayyed et al. [4]	Free flaps	95.6%
Berger et al. [5]	Local/pedicled flaps	98.1%
Grauberger et al. [6]	Free flaps	95.3%
Kim et al. [10]	Free diabetic heel flaps	73%
Ahn et al. [13]	Perforator pedicled flaps	Complete survival
Lin et al. [14]	Perforator pedicled flaps	Near-universal survival
Sarker et al. [16]	Reverse sural flap	Near-universal survival
Ciofu et al. [21]	Reverse sural flap	Complete survival

**Table 3:** Flap selection considerations derived from the studied literature.

Clinical Situation/Defect Characteristics	Commonly Reported Reconstructive Options	Supporting Studies
Small heel/foot defect, good local tissue	Locoregional/random local flaps	[1,4,6,13,16]
Plantar weight bearing heel, large defect	Free ALT, latissimus, medial plantar (sensate), other fasciocutaneous	[2,4,6,7,9,12]
Ankle/Achilles tendon soft tissue loss	Reverse sural, peroneal or anterior tibial perforator pedicled flaps, free flaps	[2,6,13,14,16]
Diabetic/neuropathic heel with poor vascularity	Free flaps after vascular optimization; medial plantar/combined reconstructive approaches	[5,10,19]
Chronic infected wounds with dead space	Free muscle flaps (particularly latissimus dorsi) are frequently reported options.	[2,8,10]

**Table 4:** Summary of main conclusions and strength of evidence based on the reviewed literature.

Claim	Evidence Strength	Supporting Studies	Reasoning
Both free and pedicled flaps achieve high flap survival, limb salvage, and satisfactory ambulation when appropriately indicated	Moderate-to-Strong	[1,2,4,5,6,13,14,16,17]	Multiple cohorts and clinical series consistently reported high flap survival and favorable limb salvage outcomes for both reconstructive strategies, even though most evidence is retrospective and heterogeneous
Large, complex, infected, or weight-bearing heel defects are frequently managed with free flaps	Moderate	[2,4,6,8,10,12,19]	Larger retrospective series and reconstructive algorithm studies predominantly used free flaps for extensive plantar defects, exposed calcaneus, osteomyelitis, major trauma, and complex diabetic wounds
Fasciocutaneous free flaps provide improved sensory recovery and earlier weight bearing compared with muscle free flaps	Moderate	[18]	Supported primarily by the meta-analysis and case series by Chellamuthu et al., which demonstrated improved sensory recovery and earlier ambulation, despite no significant differences in ulceration or flap survival were identified
Sensory recovery appears to contribute to long-term durability and may reduce ulcer-related complications.	Moderate	[1,2,7,9,18]	Several studies demonstrated improved functional durability and lower ulcer-related problems in sensate reconstructions, particularly medial plantar and nerve-coapted flaps
Ulcer recurrence remains one of the major long-term limitations after heel reconstruction	Strong	[1,2,6,12]	Multiple studies consistently reported delayed ulceration after reconstruction, mostly in weight-bearing heel regions, with ulceration rates approaching 39–41% in some cohorts
Diabetic patients with peripheral vascular disease have significantly higher complication and flap failure rates	Strong	[4,5,10,20]	Several studies identified diabetes mellitus, vascular insufficiency, renal disease, smoking, and poor nutritional status as major predictors of flap complications, flap loss, and eventual amputation
Pedicled and perforator-based flaps are reliable options for small-to-medium distal lower extremity defects	Strong	[1,5,13,14,15,16,21]	Multiple retrospective and clinical series demonstrated high survival rates, acceptable functional outcomes, preserved vascular anatomy, and relatively low donor-site morbidity
Current literature does not clearly demonstrate superiority of free versus pedicled flaps overall	Moderate	[2,5,11,15,17,18]	Direct comparative evidence remains limited because of substantial heterogeneity, selection bias, and major differences in defect complexity between reconstructive groups

## Data Availability

Data sharing is not applicable to this review as no new data were generated.
